# Speech enhancement method integrating squeeze and excitation channel attention mechanism and improved CGAN

**DOI:** 10.1371/journal.pone.0359641

**Published:** 2026-10-01

**Authors:** Yecai Guo, Meiyu Liang, Tianyue Jiang

**Affiliations:** 1 School of Electrical and Electronic Engineering, Anhui Institute of Information Technology, Wuhu, Anhui, China; 2 College of Electronic and Information Engineering, Nanjing University of Information Science and Technology, Nanjing, Jiangsu, China; Lincoln University College, MALAYSIA

## Abstract

To avoid the additional computational cost and algorithmic latency introduced by short-time Fourier transformation and waveform reconstruction, we propose CGAN-SECA, which incorporates a squeeze-and-excitation channel attention mechanism to enhance noisy speech directly in the time domain. The generator employs a symmetric encoder-decoder backbone with skip connections to reconstruct waveform samples and reduce information loss during down-sampling and up-sampling. SECA adaptively recalibrates channel-wise feature responses, enabling the network to emphasize speech-relevant representations under complex noise. In the discriminator, Virtual Batch Normalization (VBN) and Dropout are introduced to regularize adversarial training. Least-squares adversarial objectives replace cross-entropy-based objectives, and an L1 reconstruction term encourages sample-level fidelity. The method is evaluated on VoiceBank-DEMAND, which contains environmental and babble noise, and on the THCHS30-DNS Challenge dataset under multiple Signal-to-Noise Ratio (SNR) conditions. On VoiceBank-DEMAND, CGAN-SECA achieved PESQ and STOI scores of 3.018 and 0.954, respectively, ranking second only to CMGAN and outperforming the remaining comparison methods. CGAN-SECA also maintained fast inference, with a real-time factor (RTF) of 0.00423. On the THCHS30-DNS Challenge dataset, CGAN-SECA achieved the highest average PESQ and STOI scores among all evaluated methods, reaching 2.274 and 0.838, respectively, across the −5, 0, and 5 dB conditions. In particular, it produced the best results for both metrics at 0 and 5 dB, demonstrating effective speech enhancement and good generalization across different noise levels and datasets.

## Introduction

Speech enhancement refers to the process of using various signal processing and improvement technologies to heighten the quality, clarity and understandability of speech signals. As the key front-end technology of speech processing, it is a challenging task. Researchers have tried to implement various speech enhancement algorithms. Among them, traditional single-channel speech enhancement algorithms, that is, non-neural network speech enhancement algorithms, such as spectral subtraction method [1], subspace method [2], etc., belong to unsupervised learning algorithms. By assuming a stationary noise signal, these unsupervised learning algorithms are used to eliminate noises from noisy speech, thereby achieving the purpose of the speech enhancement. However, in practical applications, the noise signals are not always stable. Therefore, based on the assumption that the noise is stationary, it is very difficult for these algorithms to be used to eliminate the non-stationary noises from the noisy speech. Since their denoising effect is very poor, they cannot effectively materialize the purpose of the speech enhancement. Therefore, Speech enhancement tasks need to be tackled urgently.

In recent years, in order to deal with the non-stationary noises better, deep learning models have been introduced into speech enhancement tasks, which include two main methods. One is Mask, whose speech spectrum can fully display the time and frequency domain information. So, the Mask method starts from the speech spectrum and realizes speech enhancement via processing the time-frequency units on the spectrum [3]. Common Mask methods include: Ideal Binary Mask (IBM) [4], Ideal Ratio Mask(IRM) [5], Ideal Amplitude Mask(IAM) [6], Phase Sensitive Mask(PSM) [7], complex Ideal Ratio Mask(cIRM) [8], Accurate Ratio Masking(ARM) [9], etc. Although a good enhancement effect of the speech can be achieved by using Mask, the speech spectrum cannot be directly converted into speech signal, and it needs additional operations to convert into speech signal. The other is Mapping, which uses the deep learning models to learn the mapping relationship between the noisy speech and pure speech and may be divided into spectrum mapping [10] and waveform mapping [11].

Common deep learning models are as follows: Deep Neural Network (DNN) [12]. The DNN can be used to build nonlinear functions, but too long input data will lead to too many parameters, too short input data will result in that the model has limited ability to capture contextual dependencies, and it is difficult to capture the long-term dependence and global structural characteristics of the speech signals. Convolutional Neural Networks (CNN) can solve the problem of limited input data, but cannot make good use of the context information of speech [13]. In order to make better use of the speech information, Long-Short Term Memory (LSTM) model [14,15] is employed to realize speech enhancement. In the LSTM model, the output of the current moment depends on the information of the previous moment, and the model can make full use of the fore and aft correlation of the data. However, there are some problems in training, such as the difficulty in capturing the long-term dependence among the speech signals, gradient disappearance or gradient explosion. In order to effectively establish a long-term dependency relationship, Gated Linear Unit (GLU) is introduced into the convolutional recurrent network to establish Gated Convolutional Recurrent Networks (GCRN), but it cannot understand the context relationship between speech data very well. In order to make full use of the speech information in the speech enhancement task, Researchers [16] designed some speech enhancement algorithms based on Gate-Driven Recurrent Convolutional Neural Network (GDRCNN), Fully Convolutional Neural Networks(FCNN) [17], or Convolutional-Recurrent Neural Network (CRN) [18], etc. Many time-frequency-domain speech enhancement methods first convert speech waveforms into time-frequency representations and reconstruct the waveforms after enhancement. These operations require magnitude and phase processing and introduce additional computation and algorithmic delay. In contrast, direct waveform mapping learns the relationship between noisy and clean speech directly in the time domain, avoiding the intermediate transformation and reconstruction steps. Based on this time-domain formulation, we propose an improved Conditional Generative Adversarial Network integrating a Squeeze-and-Excitation Channel Attention mechanism (CGAN-SECA). In the proposed model, the generator adopts a symmetric encoder–decoder architecture. Both the encoder and decoder incorporate SECA modules and use PReLU activation functions. Skip connections transfer features from each encoder layer to the corresponding decoder layer, thereby reducing information loss during downsampling and supporting detailed waveform reconstruction. Unlike the generator, the discriminator is an encoder-only network that processes channel-wise concatenated waveform pairs through one-dimensional convolutional blocks. The discriminator consists of the convolutional layers, SECAs, and Virtual Batch Normalization (VBN) modules, Dropout layers that are added to prevent overfitting, an LReLU function as the activation function, and the output layer including a one-dimensional convolutional layer, a fully connected layer, and a Sigmoid function. In the CGAN, SECA directs the model toward informative speech features and adjusts the channel weights during training. This channel weighting suppresses noise-related features while preserving speech content, thereby improving clarity and intelligibility.

In summary, the main contributions of our work are as follows:

1 We propose CGAN-SECA, a waveform-domain conditional generative adversarial network for direct speech enhancement. Its generator adopts a symmetric encoder–decoder architecture with skip connections to transfer multiscale features, reduce information loss during downsampling and upsampling, and support detailed waveform reconstruction.
2 The SECA modules are incorporated into both the generator and discriminator to adaptively recalibrate channel responses, strengthen speech-relevant features, and suppress noise-related features. VBN and Dropout are further introduced into the discriminator to stabilize and regularize adversarial training and mitigate overfitting.
3 The conventional binary cross-entropy adversarial objective is replaced with a least-squares adversarial objective, and an L1 reconstruction term is included to constrain the enhanced waveform toward its paired clean target. Comprehensive experiments on VoiceBank-DEMAND and THCHS30-DNS, including baseline comparisons, ablation studies, statistical significance tests, and RTF analysis, verify the effectiveness, robustness, component contributions, and inference efficiency of CGAN-SECA.

## Overall architecture

For speech enhancement, we use a Generative Adversarial Network (GAN) to generate an enhanced speech signal G(z) that approximates the clean speech signal xas closely as possible, where zdenotes the random variable [19]. However, an unconditional GAN provides the generator only with the random variable z and no direct guidance from the noisy input, which complicates clean-speech generation and slows convergence. Conditional Generative Adversarial Network (CGAN)-based speech-enhancement methods address this limitation by conditioning the generator on noisy speech [20–22]. The principle of the CGAN model based speech enhancement method is shown in Fig 1. In Fig 1, the noisy speech $x_{c}$ denotes the input to the generator G and is used as its conditional information to achieve accurate control of the generation process. Therefore, the input of the generator G includes not only random variablez, but also the noisy speech $x_{c}$ as conditional information. At this time, the generator G can generate enhanced speech similar to pure speech based on the features of the noisy speech, such as timbre, pitch, spectral characteristics, etc, thereby suppressing noise while preserving key information of the input speech. For example, the generator Gcan generate enhanced speech with similar harmonic structure and formant characteristics by learning the spectrum distribution of the noisy speech, thus avoiding speech distortion or “over smoothing” phenomenon that may occur in traditional algorithms. In addition, the generator Gno longer needs to completely reconstruct the speech signal from random noise, but uses the guidance information from noisy speech $x_{c}$ to generate the target or pure speech more efficiently. Through this conditional control, the generator can obtain more useful information during the training process to accelerate convergence and improve training efficiency. Furthermore, it can finely control the characteristics of the generated speech to ensure that the final enhanced speech better meets the requirements of the pure speech. Since the CGAN has significant advantages in speech enhancement tasks under stationary noise environments, it can provide higher-quality speech in different noise environments, and also improve the stability and robustness of the model in practical applications. However, it still has some problems, such as gradient vanishing or pattern collapse, limited modeling ability, and restricted generalization ability under non-stationary noise environments.

**Fig 1:**
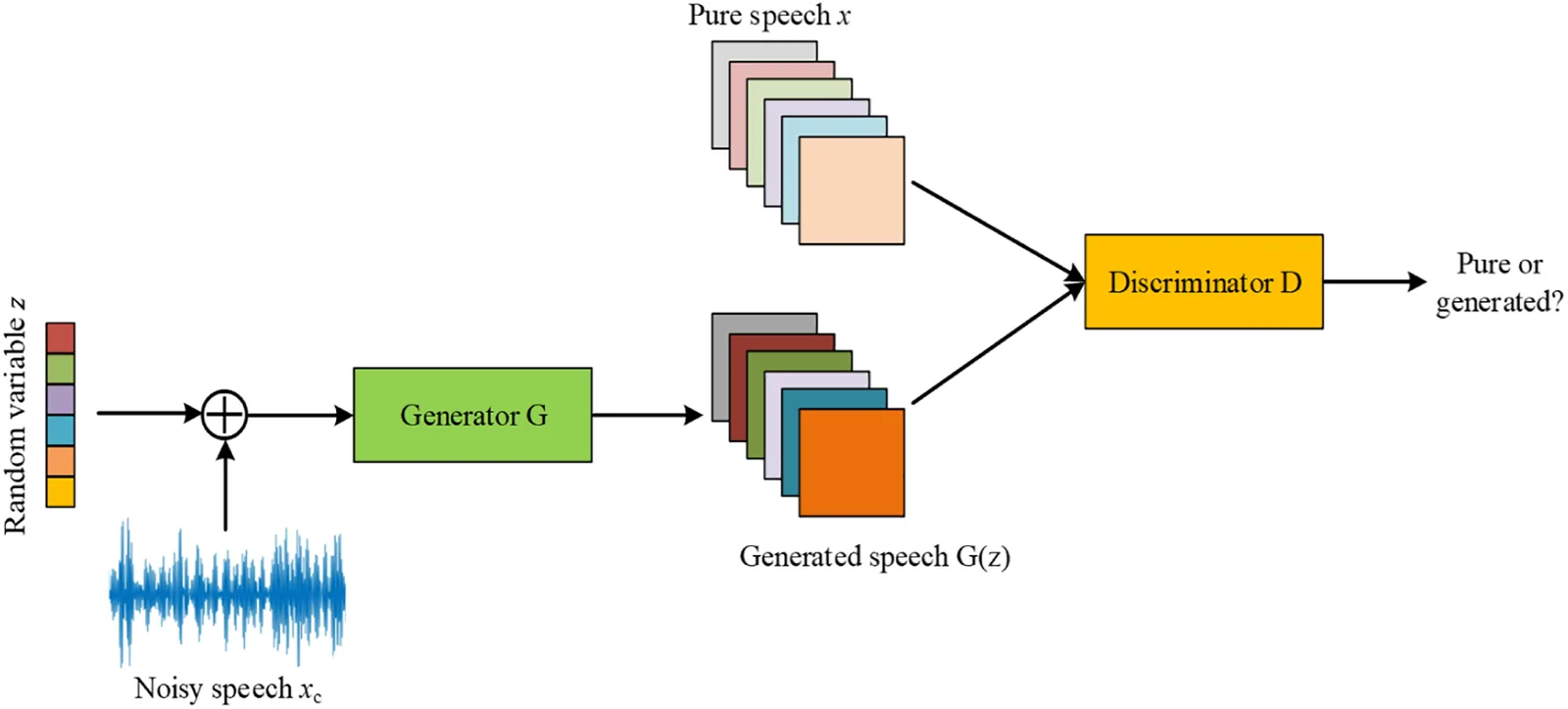
Speech enhancement process of the CGAN.

## Improvements of the modules in overall architecture

To address some existing problems in Fig 1, we have made improvements to some modules in Fig 1. to propose the Conditional Generative Adversarial Network fusing Squeeze and Excitation Channel Attention mechanism(CGAN-SECA) based speech enhancement method, as described below.

### Integration of squeeze and excitation channel attention mechanism to the CGAN

The core of channel attention mechanism is to enable the model to automatically learn to assign different importance weights to different feature channels, thereby enhancing useful features and suppressing useless features. Its basic idea is to adaptively weight each channel of the input feature map by learning a vector of channel-attention weights to achieve feature recalibration. Based on this basic idea, we introduce the channel attention mechanism [23] into deep learning model for speech enhancement to dynamically adjust the importance of each feature channel, suppress noise related feature channels, and preserve speech related feature channels, thereby improving the speech enhancement performance of the model. Specifically, the channel attention mechanism is used to calculate the weight of each feature channel according to different noise environments, then the input features are weighted using these learned coefficients to enhance effective information related to speech and suppress irrelevant features related to noise, thereby enhancing the adaptability and robustness of the model, enabling the model to perform well under different noise conditions.

In order to further improve the focus of the model on pure speech while suppressing noise features, the Squeeze and Excitation Channel Attention(SECA) mechanism is employed to enhance the expressiveness and performance of the model by making more fine-grained adjustments to the features of each channel. The structure of the SECA is shown in Fig 2. Specifically, the SECA mechanism includes two steps: Squeeze and Excitation. In the Squeeze step, the SECA is to apply a squeeze operation on each channel to compress the global information of each channel feature, so that the model can focus on learning global information. In the Excitation step, the weights of the channels are further optimized and the importance of each channel in generating speech [24] is adjusted. Therefore, the SECA can more effectively suppress noise channels and enhance the response to the speech features, thus improving the speech enhancement effect of the model.

**Fig 2:**
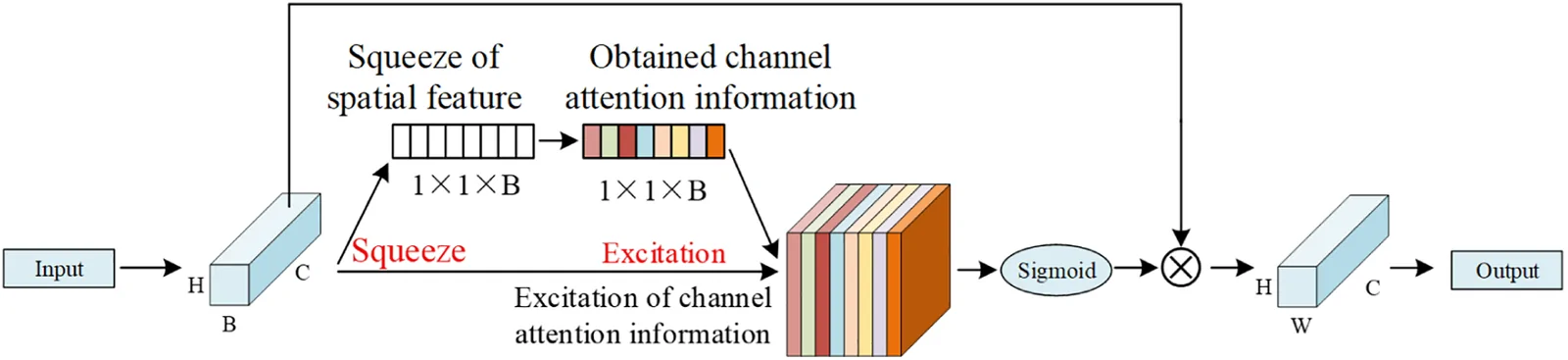
Squeeze and excitation channel attention mechanism.

In Fig 2, the input dimension of the SECA mechanism is (B,C,H), whereB is the batch size, Cmeans the number of the channels, and Hdenotes the height of the feature map (in speech enhancement, it is called the number of time steps). The squeeze part adopts adaptive average pooling. Firstly, the input feature map is compressed into the global average of each channel in the time dimension, where each channel is represented by a scalar value. At this point, the dimension of the output becomes (B,C,1). Secondly, the output with dimension (B,C,1)is flattened into the shape of (B,C) for further processing. The excitation part includes two fully connected layers that restore the number of the channels of the dimensional reduction to the original number of the channels C. Again, the Sigmoid activation function is used to compress the output between 0 and 1 to obtain the attention weight for each channel, which determines the importance of each channel in the final feature map. Finally, the weights of the channels are multiplied channel by channel and applied to the original input feature map.

Since the SECA mechanism can explicitly model the dependencies between the channels, adaptively reweight feature channels, and enhance the generalization ability of the model, it is introduced into the CGAN to result in the CGAN-SECA based speech enhancement method.

### Improvement of generator and discriminator in the CGAN

To improve CGAN performance, we refine both the generator and discriminator, the overall architecture is shown in Fig 1**.** The improved generator is shown in Fig 3.

**Fig 3:**
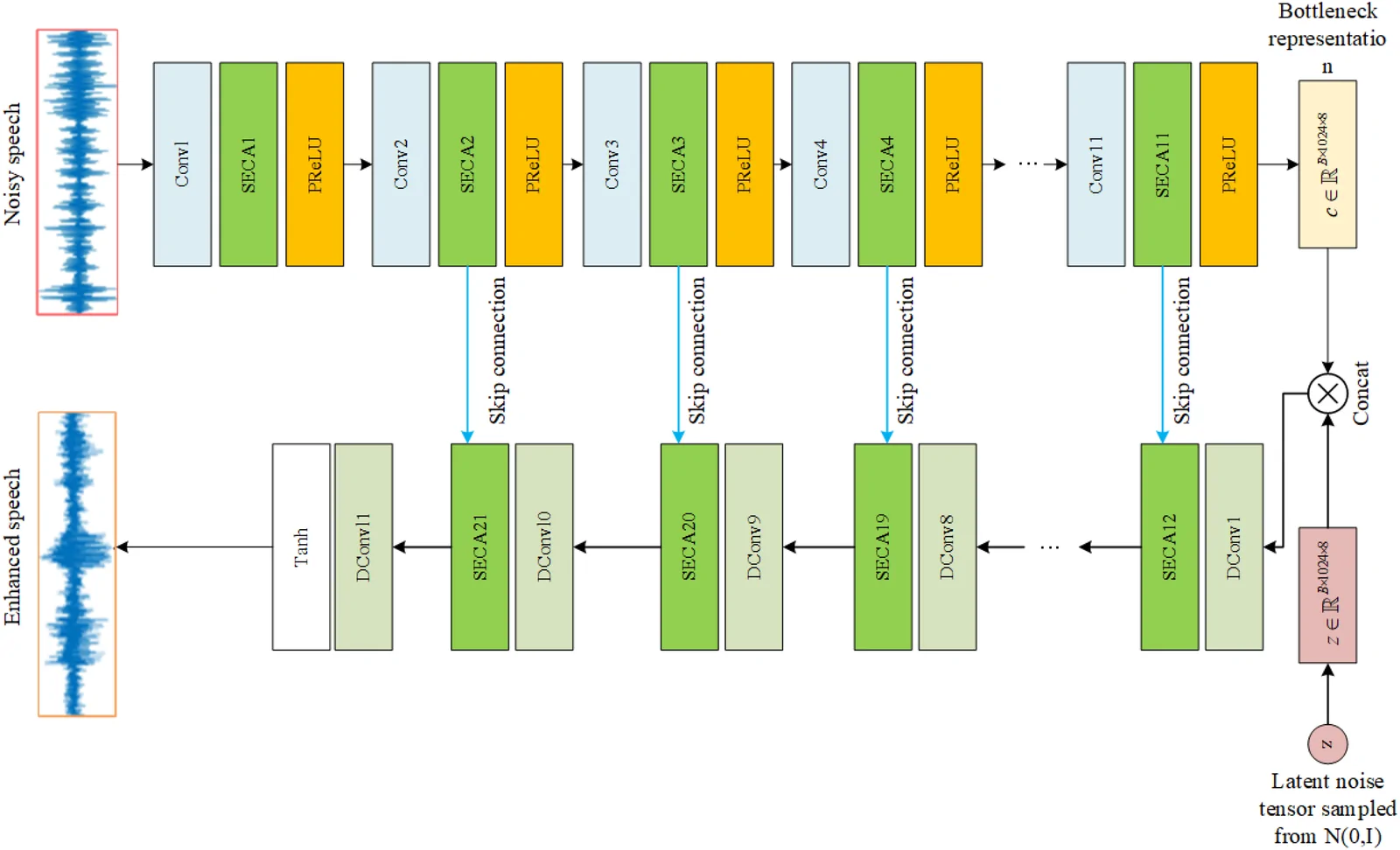
Network structure of the improved generator.

In Fig 3, the generator adopts a symmetric encoder-decoder structure. The encoder contains eleven convolutional layers, each followed by a SECA module and a PReLU activation. Its bottleneck representation is $c\in {\mathbb{R}}^{B\times 1024\times 8}$. A latent tensor $z\in {\mathbb{R}}^{B\times 1024\times 8}$is sampled independently from a standard normal distribution N(0,I) for every minibatch during training and for every segment during inference. After z and c are concatenated along the channel dimension, the decoder input has shape B×2048×8. The decoder is symmetric to the encoder and contains eleven transposed-convolutional layers, each followed by a SECA module. Skip connections reuse the corresponding encoder features to support detailed waveform reconstruction. Finally, the Tanh layer limits the enhanced waveform to (−1,1). Table 1 shows the input-output dimensions of each layer in the generator and the parameter settings of the convolutional layers. k, s, p, and crepresent the size of the convolutional kernel, the stride, padding, and the number of channels, respectively. The number of input feature channels in each decoding layer doubles due to skip connections.

**Table 1:** Network parameters of the generator.

	Network layer	Input dimension	Output dimension	Parameters
**Encoding end**	Conv1	B × 1 × 16384	B × 16 × 8192	k=32,s=2,p=15
	SECA1	B × 16 × 8192	B × 16 × 8192	C=16
	Conv2	B × 16 × 8192	B × 32 × 4096	k=32,s=2,p=15
	SECA2	B × 32 × 4096	B × 32 × 4096	C=32
	Conv3	B × 32 × 4096	B × 32 × 2048	k=32,s=2,p=15
	SECA3	B × 32 × 2048	B × 32 × 2048	C=32
	Conv4	B × 32 × 2048	B × 64 × 1024	k=32,s=2,p=15
	SECA4	B × 64 × 1024	B × 64 × 1024	C=64
	Conv5	B × 64 × 1024	B × 64 × 512	k=32,s=2,p=15
	SECA5	B × 64 × 512	B × 64 × 512	C=64
	Conv6	B × 64 × 512	B × 128 × 256	k=32,s=2,p=15
	SECA6	B × 128 × 256	B × 128 × 256	C=128
	Conv7	B × 128 × 256	B × 128 × 128	k=32,s=2,p=15
	SECA7	B × 128 × 128	B × 128 × 128	C=128
	Conv8	B × 128 × 128	B × 256 × 64	k=32,s=2,p=15
	SECA8	B × 256 × 64	B × 256 × 64	C=256
	Conv9	B × 256 × 64	B × 256 × 32	k=32,s=2,p=15
	SECA9	B × 256 × 32	B × 256 × 32	C=256
	Conv10	B × 256 × 32	B × 512 × 16	k=32,s=2,p=15
	SECA10	B × 512 × 16	B × 512 × 16	C=512
	Conv11	B × 512 × 16	B × 1024 × 8	k=32,s=2,p=15
	SECA11	B × 1024 × 8	B × 1024 × 8	C=1024
**Decoding end**	Deconv1	B × 2048 × 8	B × 512 × 16	k=32,s=2,p=15
	SECA12	B × 512 × 16	B × 512 × 16	C=512
	Deconv2	B × 1024 × 16	B × 256 × 32	k=32,s=2,p=15
	SECA13	B × 256 × 32	B × 256 × 32	C=256
	Deconv3	B × 512 × 32	B × 256 × 64	k=32,s=2,p=15
	SECA14	B × 256 × 64	B × 256 × 64	C=256
	Deconv4	B × 512 × 64	B × 128 × 128	k=32,s=2,p=15
	SECA15	B × 128 × 128	B × 128 × 128	C=128
	Deconv5	B × 256 × 128	B × 128 × 256	k=32,s=2,p=15
	SECA16	B × 128 × 256	B × 128 × 256	C=128
	Deconv6	B × 256 × 256	B × 64 × 512	k=32,s=2,p=15
	SECA17	B × 64 × 512	B × 64 × 512	C=64
	Deconv7	B × 128 × 512	B × 64 × 1024	k=32,s=2,p=15
	SECA18	B × 64 × 1024	B × 64 × 1024	C=64
	Deconv8	B × 128 × 1024	B × 32 × 2048	k=32,s=2,p=15
	SECA19	B × 32 × 2048	B × 32 × 2048	C=32
	Deconv9	B × 64 × 2048	B × 32 × 4096	k=32,s=2,p=15
	SECA20	B × 32 × 4096	B × 32 × 4096	C=32
	Deconv10	B × 64 × 4096	B × 16 × 8192	k=32,s=2,p=15
	SECA21	B × 16 × 8192	B × 16 × 8192	C=16
	Deconv11	B × 32 × 8192	B × 1 × 16384	k=32,s=2,p=15
	Tanh	B × 1 × 16384	B × 1 × 16384	——

Unlike the generator, the discriminator [25] only have the process of one-dimensional convolutional encoding and feature concatenation layer by layer, without a decoding part [26]. Feature concatenation is performed on the channel dimension to fuse feature information from different levels and enhance the discriminative ability of the discriminator. The structure of discriminator is shown in Fig 4.

**Fig 4:**
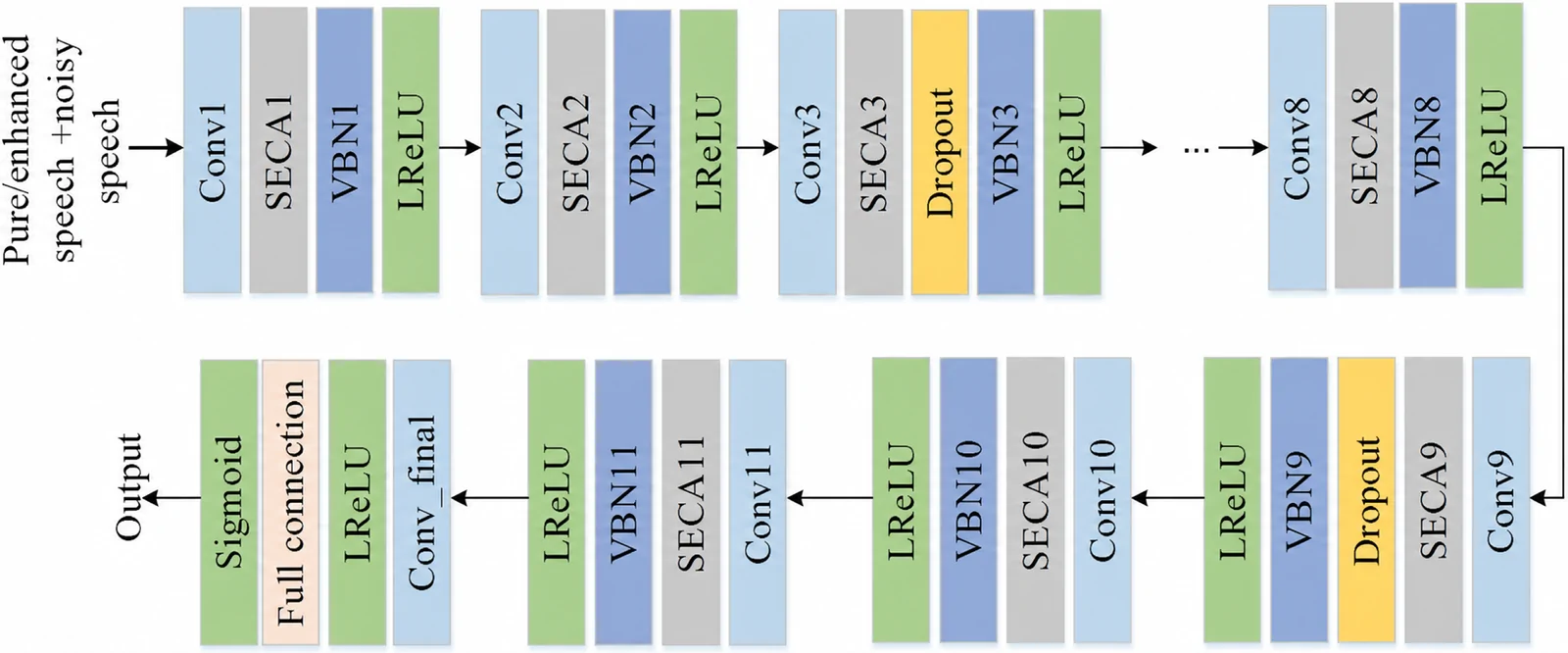
Structure of the improved discriminator.

In Fig 4, the discriminator receives a tensor of shape B×2×16384. The two channels are concatenated waveform pairs: the clean target and its noisy condition for real samples, or the generated enhanced waveform and the same noisy condition for fake samples. It consists of eleven convolutional layers, each followed by SECA and a Virtual Batch Normalization (VBN) layer. Dropout is inserted after the third, sixth, and ninth convolutional blocks. The final one-dimensional convolution, LReLU activation, fully connected layer, and Sigmoid function produce a score in (0,1), interpreted as the probability that the input pair is real. Table 2 shows the input-output dimensions of each layer in the discriminator and the parameter settings of the convolutional layers. krepresents the size of the convolutional kernel, s denotes the stride, pstands for padding, and c is the number of channels.

**Table 2:** Network parameters of the discriminator.

Network layer	Input dimension	Output dimension	Parameters
**Conv1**	B × 2 × 16384	B × 32 × 8192	k=31,s=2,p=15
**SECA1**	B × 32 × 8192	B × 32 × 8192	C=32
**VBN1**	B × 32 × 8192	B × 32 × 8192	C=32
**Conv2**	B × 32 × 8192	B × 64 × 4096	k=31,s=2,p=15
**SECA2**	B × 64 × 4096	B × 64 × 4096	C=64
**VBN2**	B × 64 × 4096	B × 64 × 4096	C=64
**Conv3**	B × 64 × 4096	B × 64 × 2048	k=31,s=2,p=15
**SECA3**	B × 64 × 2048	B × 64 × 2048	C=64
**Dropout1**	B × 64 × 2048	B × 64 × 2048	——
**VBN3**	B × 64 × 2048	B × 64 × 2048	C=64
**Conv4**	B × 64 × 2048	B × 128 × 1024	k=31,s=2,p=15
**SECA4**	B × 128 × 1024	B × 128 × 1024	C=128
**VBN4**	B × 128 × 1024	B × 128 × 1024	C=128
**Conv5**	B × 128 × 1024	B × 128 × 512	k=31,s=2,p=15
**SECA5**	B × 128 × 512	B × 128 × 512	C=128
**VBN5**	B × 128 × 512	B × 128 × 512	C=128
**Conv6**	B × 128 × 512	B × 256 × 256	k=31,s=2,p=15
**SECA6**	B × 256 × 256	B × 256 × 256	C=256
**Dropout2**	B × 256 × 256	B × 256 × 256	——
**VBN6**	B × 256 × 256	B × 256 × 256	C=256
**Conv7**	B × 256 × 256	B × 256 × 128	k=31,s=2,p=15
**SECA7**	B × 256 × 128	B × 256 × 128	C=256
**VBN7**	B × 256 × 128	B × 256 × 128	C=256
**Conv8**	B × 256 × 128	B × 512 × 64	k=31,s=2,p=15
**SECA8**	B × 512 × 64	B × 512 × 64	C=512
**VBN8**	B × 512 × 64	B × 512 × 64	C=512
**Conv9**	B × 512 × 64	B × 512 × 32	k=31,s=2,p=15
**SECA9**	B × 512 × 32	B × 512 × 32	C=512
**Dropout3**	B × 512 × 32	B × 512 × 32	——
**VBN9**	B × 512 × 32	B × 512 × 32	C=512
**Conv10**	B × 512 × 32	B × 1024 × 16	k=31,s=2,p=15
**SECA10**	B × 1024 × 16	B × 1024 × 16	C=1024
**VBN10**	B × 1024 × 16	B × 1024 × 16	C=1024
**Conv11**	B × 1024 × 16	B × 2048 × 8	k=31,s=2,p=15
**SECA11**	B × 2048 × 8	B × 2048 × 8	C=2048
**VBN11**	B × 2048 × 8	B × 2048 × 8	C=2048
**Conv_final**	B × 2048 × 8	B × 1 × 8	k=1,s=1
**Linear**	B × 1 × 8	B × 1	——

### Improved loss function

The main drawbacks of the cross-entropy loss function in the GANs are as follows [27]: (1) Unstable training, which can easily lead to mode collapse; (2) Gradient vanishing problem, when the discriminator is too confident, the generator cannot obtain effective gradients; (3) Lack of direct correlation with generation quality. Accordingly, for speech enhancement, it is necessary to improve the loss function of the GANs. In general, the loss function of the GANs is given by:


$$\begin{array}{c} \underset{G}{min\;}\underset{D}{max}\;L\text{oss}\left(D,\;G\right)={𝔼}_{x\;p_{data}\left(x\right)}\left[logD\left(x\right)\right]+{𝔼}_{z\;p_{z}\left(z\right)}\left[log\left(1-D\left(G\left(z\right)\right)\right)\right] \end{array}$$ (1)

Where $x\;p_{\text{data}}\left(x\right)$ denotes a clean-speech sample drawn from the real-data distribution, and $z\;p_{z}\left(z\right)$ denotes a latent variable sampled from a predefined prior distribution. G denotes the generator, and G(z) denotes the speech sample generated by G from the latent variable z. D denotes the discriminator, and D(·) denotes its output for a given input. Therefore, D(G(z)) represents the discriminator output for the generated sample G(z). 𝔼 denotes mathematical expectation. During adversarial training, the discriminator is optimized to assign high scores to real samples and low scores to generated samples, whereas the generator is optimized to produce samples that the discriminator classifies as real. At the beginning of training, the generated samples can be readily distinguished from real samples. As the generator improves, this distinction becomes progressively more difficult, and the two networks gradually approach an adversarial equilibrium. Accordingly, the discriminator and generator loss functions are defined as follows:


$$\begin{array}{c} L\text{oss}\left(D\right)=-{𝔼}_{x\;p_{data}\left(x\right)}\left[logD\left(x\right)\right]-{𝔼}_{z\;p_{z}\left(z\right)}\left[log\left(1-D\left(G\left(z\right)\right)\right)\right] \end{array}$$ (2)

$$\begin{array}{c} L\text{oss}\left(G\right)=-{𝔼}_{z\;p_{z}\left(z\right)}\left[log\left(D\left(G\left(z\right)\right)\right)\right] \end{array}$$ (3)

Equations (2) and (3) use the cross entropy loss function as the objective function of the GAN, which utilizes Jensen-Shannon (JS) divergence for training network. However, when the distribution of the generated samples does not overlap with that of the real samples, the JS divergence reaches a constant value. Therefore, the gradient of the objective function of the generator G relative to its parameters is 0, resulting in the problem of gradient vanishing. In addition, in order to deceive the discriminator D, the generator G may tend to generate samples of a single category, a situation known as mode collapse. SEGAN has also been applied to ego-noise attenuation in speech sound source localization, demonstrating the applicability of waveform-domain adversarial enhancement to task-specific noise conditions [28].

To mitigate vanishing gradients and mode collapse, we adopt the Least Squares Generative Adversarial Network (LSGAN) objective introduced by Mao et al. [29]. Its loss function is represented as:

Related conditional GAN formulations provide useful context. Pix2Pix combines a paired conditional adversarial loss with an L1 reconstruction term for image-to-image translation [30], whereas SEGAN performs end-to-end waveform speech enhancement with a GAN architecture [31]. The present method likewise uses paired waveform conditioning, but differs by combining an LSGAN objective with SECA, VBN, and Dropout in the speech-enhancement generator-discriminator framework.


$$\begin{array}{c} \text{min }L\text{oss}V_{LSGAN}(D)=\frac{\text{1}}{\text{2}}{𝔼}_{x\sim P_{data}(x)}\left[{\left(D(x)-b\right)}^{\text{2}}\right]+\frac{\text{1}}{\text{2}}{𝔼}_{z\sim P_{z}(z)}\left[{\left(D\left(G(z)\right)-a\right)}^{\text{2}}\right] \end{array}$$ (4)

$$\begin{array}{c} \text{min }L\text{oss}V_{LSGAN}(G)=\frac{\text{1}}{\text{2}}E_{z\sim P_{z}(z)}\left[{\left(D\left(G(z)\right)-d\right)}^{\text{2}}\right] \end{array}$$ (5)

In Equations (4) and (5), a and b denote the target labels assigned to generated and real samples, respectively, while d denotes the target label used to optimize the generator. In this study, a=0, b=1, and d=1. When the generator G is fixed, the optimal discriminator is given by:


$$\begin{array}{c} D_{opt}\left(x\right)\text{=}\frac{bp_{data}\left(x\right)+ap_{g}\left(x\right)}{p_{data}\left(x\right)+p_{g}\left(x\right)} \end{array}$$ (6)

After introducing additional condition information $x_{c}$ in the GAN, the loss function in Equation (1) is modified as [22]


$$\begin{array}{c} {min}_{G}{max}_{D}V\left(D,G\right)={𝔼}_{\left(x,x_{c}\right)\;p_{\text{data}},\text{\textbackslash{}hspace\{0.5em}}z\;𝒩\left(0,I\right)\}\left[logD\left(x,x_{c})+log(1-D\left(G\left(z,x_{c}\right),x_{c}\right)\right)\right] \end{array}$$ (7)

Equation (7) is called the loss function of the CGAN, and it is similar to the cross entropy loss function. We modify Equations (4) and (5) as:


$$\begin{array}{c} {\text{min}}_{D}L_{\text{LSGAN}}^{V}(D)=\frac{\text{1}}{\text{2}}E_{\left(x,x_{c}\right)\sim P_{\text{data}}}\left[{\left(D\left(x,x_{c}\right)-\text{1}\right)}^{\text{2}}\right]+\frac{\text{1}}{\text{2}}E_{x_{c}\sim P_{\text{data}}\left(x_{c}\right),z\sim N(\text{0},\text{1})}\left[{\left(D\left(G\left(z,x_{c}\right),x_{c}\right)-\text{0}\right)}^{\text{2}}\right] \end{array}$$ (8)

$$\begin{array}{c} {\text{min}}_{G}L_{\text{LSGAN}}^{V}(G)={𝔼}_{\left(x,x_{c}\right)\sim P_{\text{data}},z\sim 𝒩(\text{0},\text{1})}\left[\frac{\text{1}}{\text{2}}{\left(D\left(G\left(z,x_{c}\right),x_{c}\right)-\text{1}\right)}^{\text{2}}+\lambda ||G\left(z,x_{c}\right)-{x||}_{\text{1}}\right] \end{array}$$ (9)

Here, $\left(x,x_{c}\right)\;p_{\text{data}}$ denotes a paired clean–noisy speech sample. The latent tensor z 𝒩(0,I), whereIdenotes the identity matrix, has shape B×1024×8 and is sampled independently for every training minibatch and every inference segment. λ denotes the L1-loss weight set to 100, while $∥\cdot |{|}_{\text{1}}$denotes the L1 norm. The discriminator uses real = 1 and fake = 0 targets, whereas the generator is optimized toward the real target 1. Equation (9) combines the generator adversarial term and paired L1 reconstruction term under the same joint expectation.

The training process of the proposed CGAN-SECA method using the improved CGAN is shown in Fig 5 and comprises three steps. First, real clean-noisy pairs are used to train discriminator D toward the real target. Second, generated-noisy pairs are used to train D toward the fake target. Third, D is held fixed while generator G is updated so that generated-noisy pairs approach the real target. After repeated training, G is used for speech enhancement.

**Fig 5:**
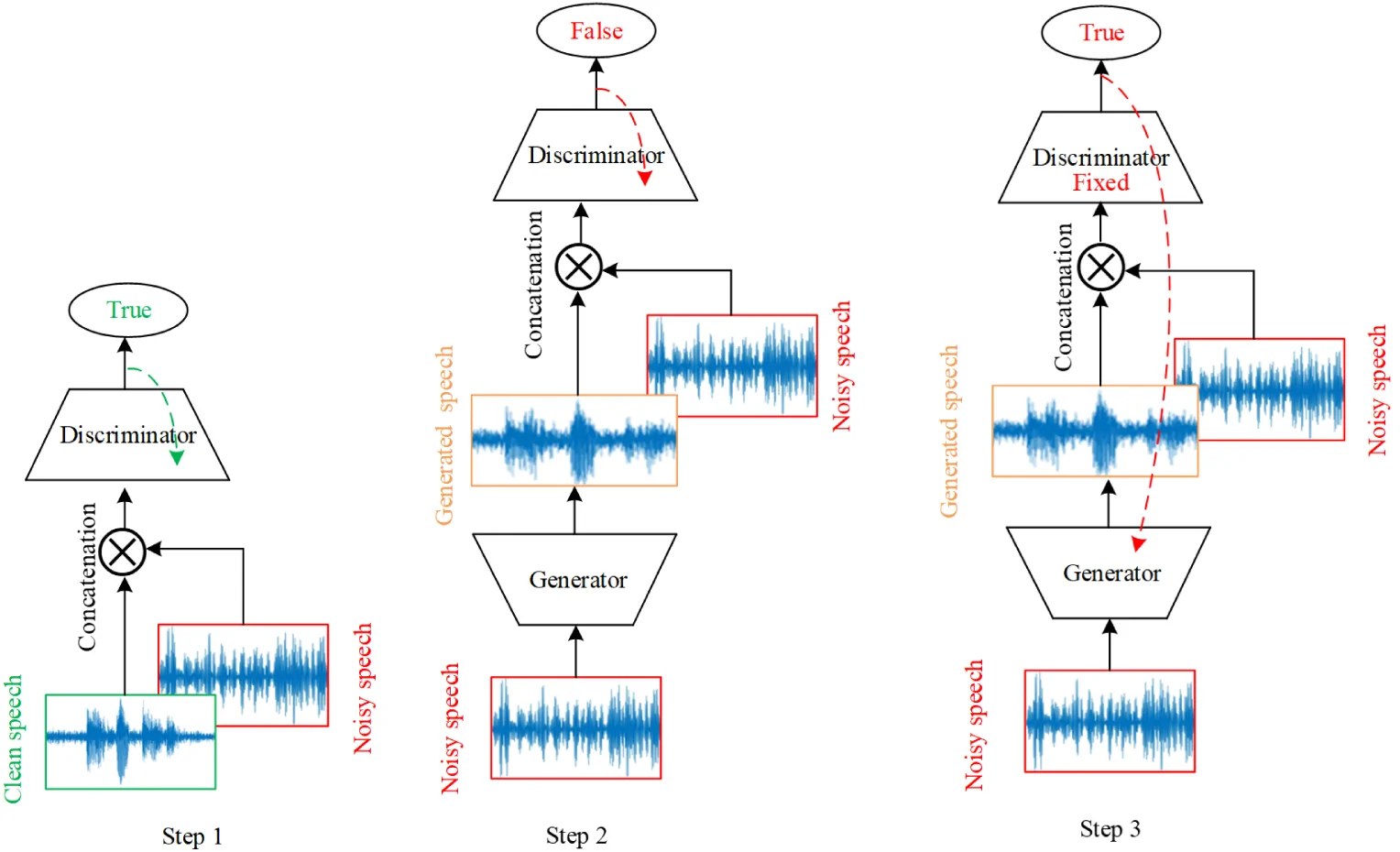
Training process of the CGAN.

## Results

### Datasets and experimental setup

VoiceBank-DEMAND and the THCHS30-DNS Challenge dataset were used to evaluate CGAN-SECA under different noise and Signal-to-Noise Ratio (SNR) conditions. VoiceBank-DEMAND pairs clean speech from the VoiceBank corpus with environmental recordings from the DEMAND database, and babble noise [32]. The training mixtures have SNRs of 0, 5, 10, and 15 dB, whereas the test mixtures have SNRs of 2.5, 7.5, 12.5, and 17.5 dB. The THCHS30-DNS Challenge dataset combines Chinese speech from THCHS30 with noises selected from the DNS Challenge noise set. The mixtures were generated at SNRs of −5, 0, and 5 dB.All waveforms were sampled at 16 kHz and processed using pre-emphasis with a coefficient of 0.95. Each waveform was divided into 16,384-sample segments using a segment shift of 8,192 samples. The generator and discriminator were optimized using RMSprop with a learning rate of $2\times 10^{-4}$for 100 epochs. The batch size was 64, and the VBN reference batch contained 128 samples. The L1 reconstruction loss weight was set to 100. All experiments were conducted on an NVIDIA GeForce RTX 3080 Ti GPU using PyTorch 2.5.1 and CUDA 11.8. PESQ and STOI were used to quantify perceptual speech quality and intelligibility.

### Comparative results on the VoiceBank-DEMAND and THCHS30-DNS challenge datasets

To evaluate the proposed CGAN-SECA, we compared it with a generative adversarial network (GAN), a conditional generative adversarial network (CGAN), the self-attention GAN for speech enhancement (SAGAN) [33], GDRCNN-ARM [16], CleanUNet [34], MetricGAN+ [35], and CMGAN [36]. The GAN generator employs a symmetric encoder–decoder architecture composed of stacked convolutional and transposed-convolutional layers, with skip connections used to transfer encoded features, while its discriminator consists of multiple convolutional layers. CGAN follows the same backbone but introduces noisy speech as conditional information for both the generator and the discriminator during adversarial training. SAGAN incorporates self-attention into the generator to improve the modeling of long-range dependencies in speech signals. GDRCNN-ARM consists of an encoder, an intermediate module, and a decoder, combining dilated convolutions, gated recurrent units, multi-head attention, layer-wise decoding, and skip connections for speech feature reconstruction. CleanUNet is a causal waveform-domain U-Net augmented with self-attention. MetricGAN+ employs an improved metric discriminator to guide the generator toward perceptual quality metrics directly. CMGAN combines Conformer-based time–frequency modeling with metric-guided adversarial learning and estimates both magnitude and complex spectral components. All models were retrained locally under the same experimental configuration.

The PESQ and STOI values obtained by different speech enhancement methods on the VoiceBank-DEMAND dataset are shown in Table 3.

**Table 3:** Comparison of CGAN-SECA with other methods on the VoiceBank-DEMAND dataset.

Methods	PESQ	STOI
**Noisy**	1.965	0.907
**GAN**	2.461	0.921
**CGAN**	2.637	0.928
**SAGAN**	2.924	0.936
**GDRCNN-ARM**	2.926	0.948
**CleanUNet**	2.957	0.950
**MetricGAN+**	3.004	0.952
**CMGAN**	3.146	0.956
**CGAN-SECA (ours)**	3.018	0.954

The PESQ and STOI results presented in Table 3 are further visualized in Fig 6 to provide an intuitive comparison among the evaluated methods.

**Fig 6:**
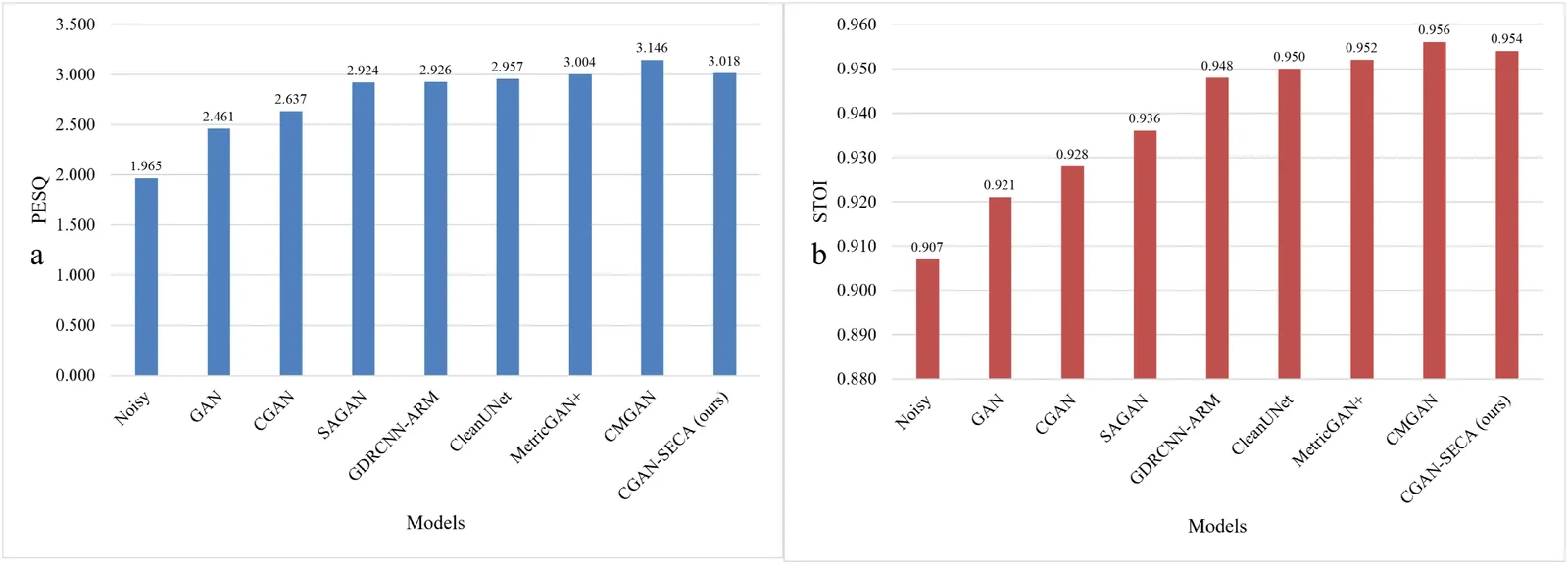
Comparison between CGAN-SECA and other methods on the VoiceBank-DEMAND dataset. (a) PESQ (b) STOI.

The PESQ and STOI values and their average values obtained by the experiments of different methods on the THCHS30-DNS Challenge dataset are shown in Table 4.

**Table 4:** Comparison between CGAN-SECA and other methods on the THCHS30-DNS Challenge dataset.

Indicators	SNRs(dB)	Noisy	GAN	CGAN	SAGAN	GDRCNN-ARM	CleanUNet	MetricGAN+	CMGAN	CGAN-SECA (ours)
**PESQ**	−5	1.048	1.507	1.613	1.562	1.661	1.674	1.711	1.852	1.768
	0	1.083	1.871	1.853	1.961	1.868	2.083	2.137	2.276	2.316
	5	1.156	2.214	2.259	2.435	2.423	2.503	2.558	2.661	2.737
**Average**	—	1.096	1.864	1.908	1.986	1.984	2.087	2.135	2.263	2.274
**STOI**	−5	0.561	0.701	0.727	0.746	0.733	0.752	0.757	0.772	0.761
	0	0.614	0.791	0.815	0.822	0.782	0.831	0.850	0.846	0.863
	5	0.662	0.836	0.847	0.853	0.831	0.860	0.877	0.873	0.891
**Average**	—	0.612	0.776	0.796	0.807	0.782	0.814	0.833	0.830	0.838

Table 3 and Table 4 present the comparative results on the VoiceBank-DEMAND and THCHS30-DNS Challenge datasets, respectively. As shown in Table 3, all evaluated speech-enhancement methods improved the noisy speech on VoiceBank-DEMAND. CGAN-SECA achieved PESQ and STOI scores of 3.018 and 0.954, representing absolute improvements of 1.053 and 0.047 over the unprocessed noisy speech, respectively. It also outperformed GAN, CGAN, SAGAN, GDRCNN-ARM, CleanUNet, and MetricGAN+ on both metrics. In particular, compared with the conventional CGAN, CGAN-SECA improved PESQ from 2.637 to 3.018 and STOI from 0.928 to 0.954, demonstrating the effectiveness of the incorporated architectural and objective-function improvements. Although CMGAN obtained the highest scores of 3.146 and 0.956, CGAN-SECA achieved the second-best results, with a STOI difference of only 0.002, indicating strong competitiveness in perceptual speech quality and intelligibility.

Table 4 further evaluates the methods under the more challenging SNR conditions of the THCHS30-DNS Challenge dataset. The performance of all methods generally increased with the SNR. CGAN-SECA achieved the highest PESQ and STOI scores at both 0 and 5 dB, reaching 2.316/0.863 and 2.737/0.891, respectively. Across the three SNR conditions, it obtained the highest average PESQ and STOI scores of 2.274 and 0.838, corresponding to improvements of 1.178 and 0.226 over the noisy speech. Compared with CMGAN, CGAN-SECA improved the average PESQ and STOI by 0.011 and 0.008, respectively. Although CMGAN performed better under the extremely challenging −5 dB condition, CGAN-SECA showed clearer advantages at 0 and 5 dB, resulting in superior overall average performance. The different relative performance of CMGAN across the two datasets may be associated with differences in speech language, noise composition, and SNR range. These results indicate that CGAN-SECA not only remains highly competitive on VoiceBank-DEMAND but also exhibits strong robustness and generalization under the diverse noise and SNR conditions of THCHS30-DNS.

Fig 7 and Fig 8 compare the PESQ and STOI of all evaluated methods on the THCHS30-DNS Challenge dataset under different SNR conditions in the form of bar charts.

**Fig 7:**
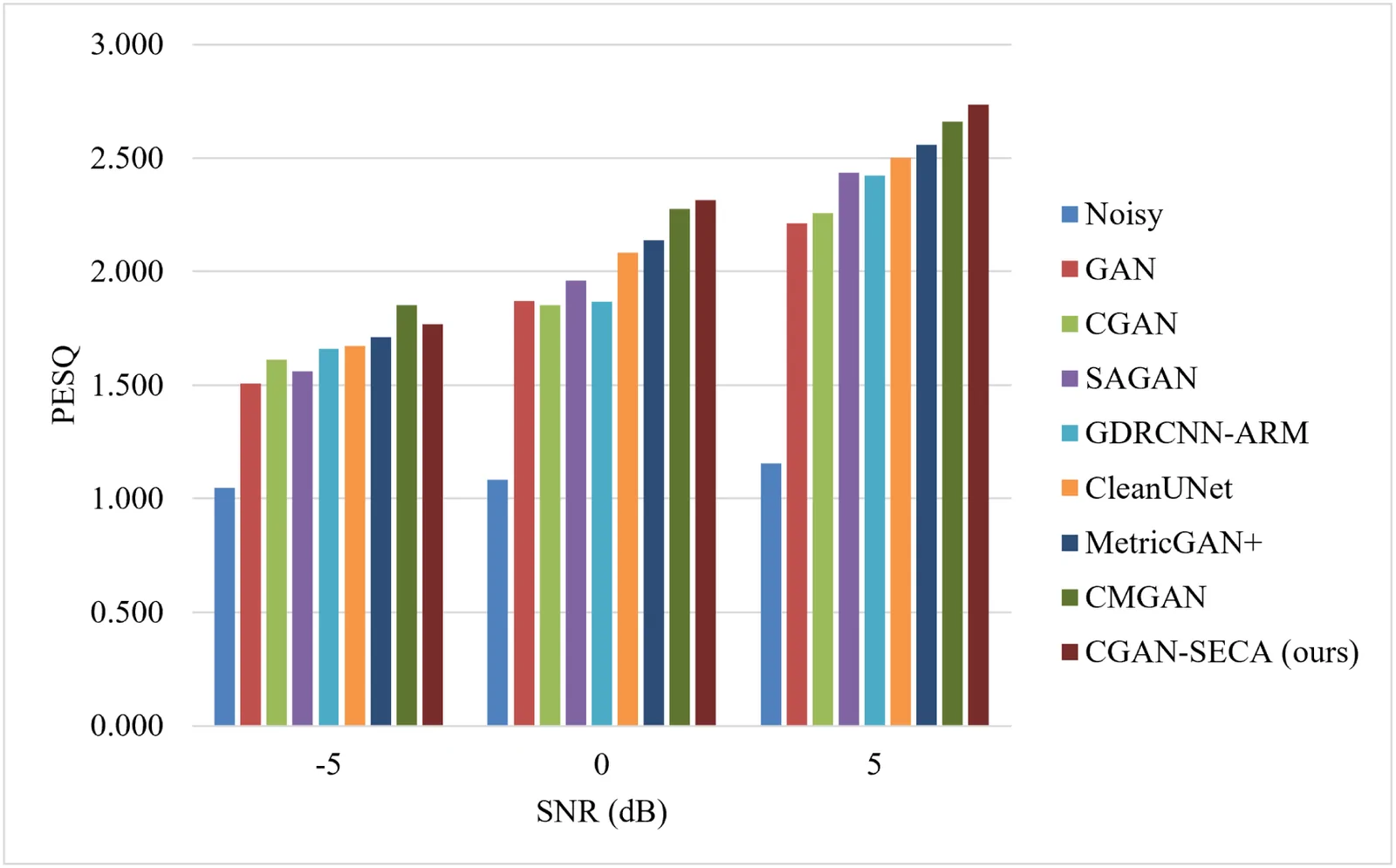
PESQ comparison of CGAN-SECA with other methods on the THCHS30-DNS Challenge dataset.

**Fig 8:**
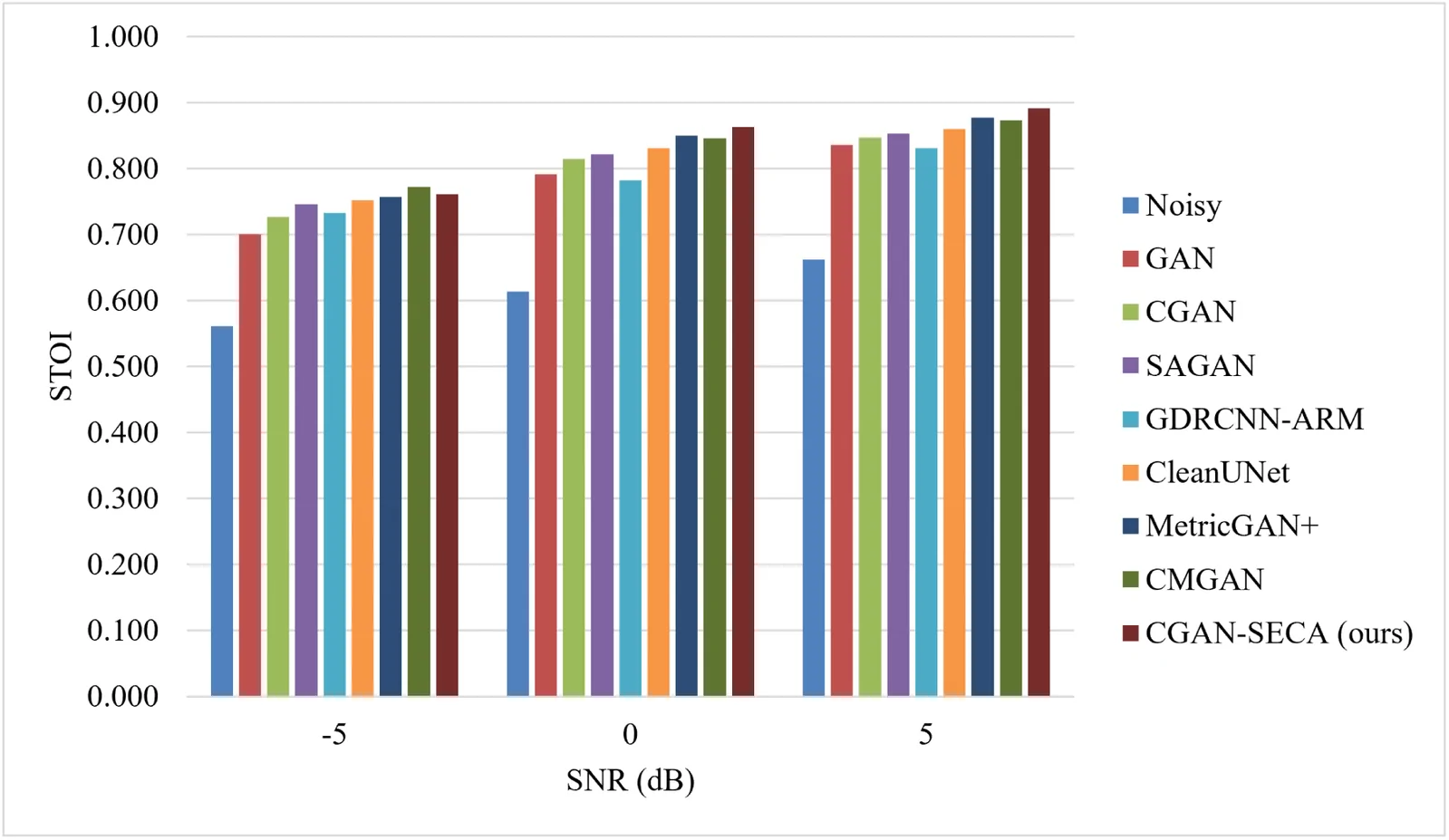
STOI comparison of CGAN-SECA with other methods on the THCHS30-DNS Challenge dataset.

### Qualitative visualization analysis

The waveform and spectrogram comparisons of different speech enhancement methods on the VoiceBank-DEMAND and THCHS30-DNS Challenge datasets are shown in Fig 9 and Fig 10, In both datasets, CGAN-SECA effectively suppresses the diffuse background-noise components while preserving the principal temporal envelope of the speech signal. Its enhanced spectrograms exhibit clearer speech-active regions and more distinct spectral structures, particularly in the speech-dominant low- and mid-frequency bands. These qualitative results are consistent with the PESQ and STOI results in Tables 3 and Tables 4, further demonstrating the effectiveness and robustness of CGAN-SECA under different speech, noise, and SNR conditions.

**Fig 9:**
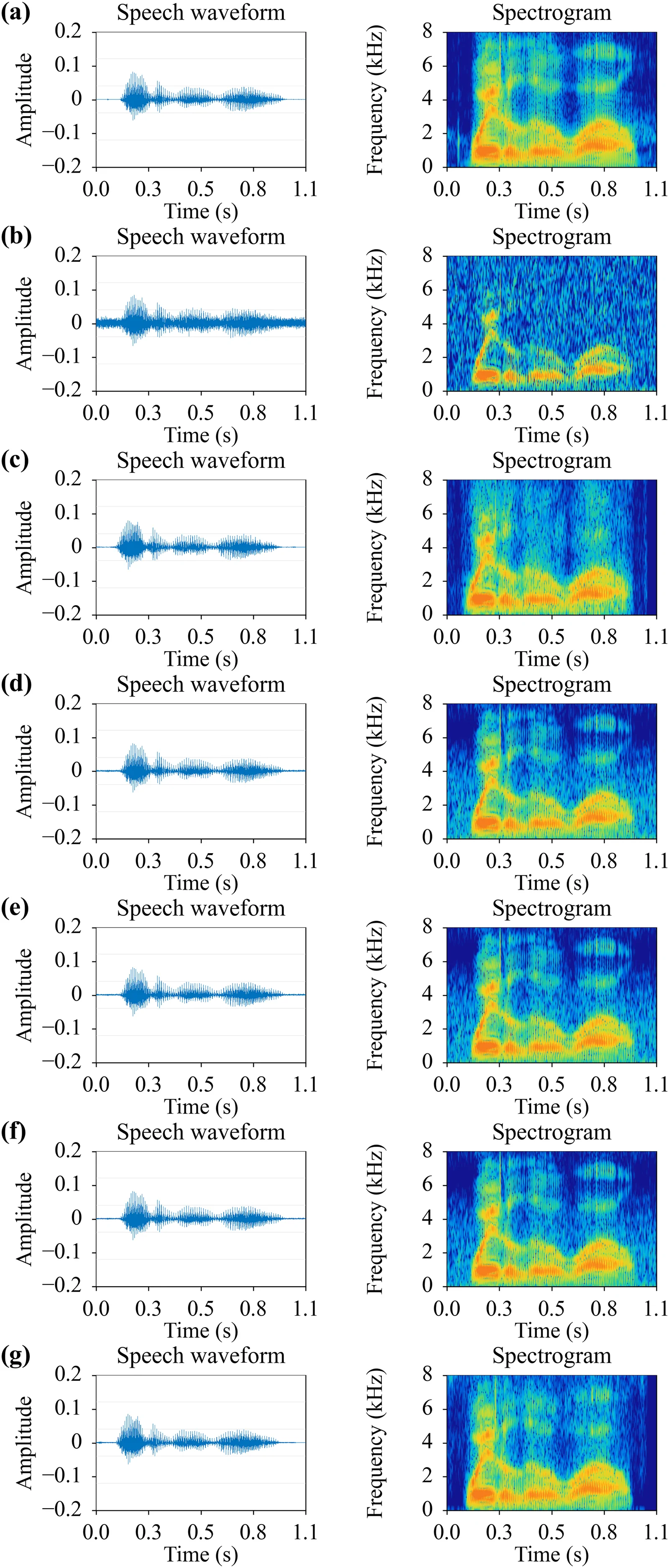
Comparison of waveforms and spectrograms under a representative noisy condition from VoiceBank-DEMAND. (a) clean speech; (b) noisy speech; (c) speech enhanced by GDRCNN-ARM; (d) speech enhanced by CleanUNet; (e) speech enhanced by MetricGAN+; (f) speech enhanced by CMGAN; and (g) speech enhanced by CGAN-SECA.

**Fig 10:**
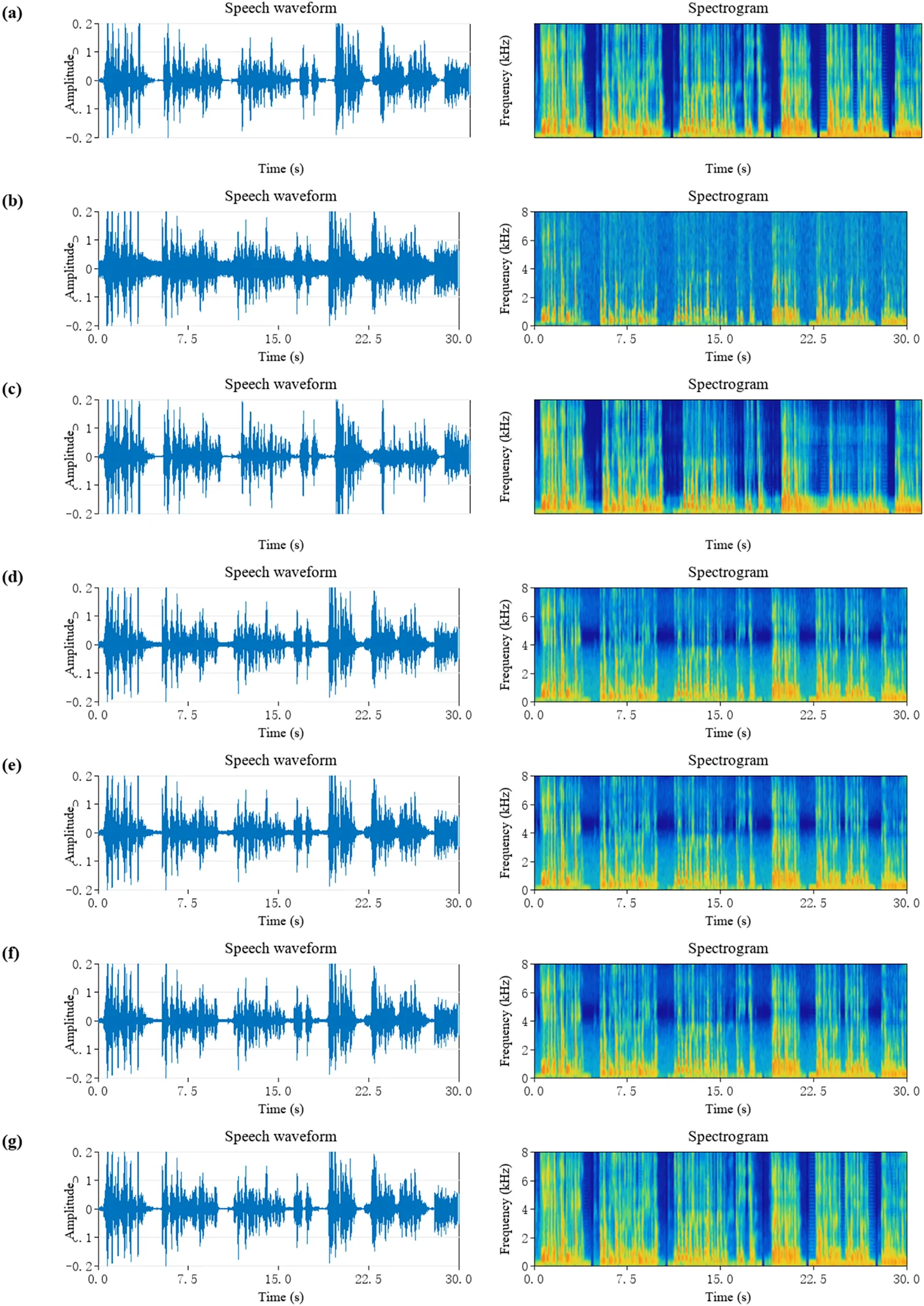
Comparison of waveforms and spectrograms under workshop noise from the THCHS30-DNS Challenge dataset. (a) clean speech; (b) noisy speech; (c) speech enhanced by GDRCNN-ARM; (d) speech enhanced by CleanUNet; (e) speech enhanced by MetricGAN+; (f) speech enhanced by CMGAN; and (g) speech enhanced by CGAN-SECA.

### Model complexity and inference efficiency analysis

Table 5 compares the parameter counts and RTFs of CGAN-SECA and the comparison methods.

**Table 5:** Comparison of parameter counts and real-time factors among CGAN-SECA and the comparison methods.

Methods	Parameters(×10^6^)	RTF↓
**GAN**	75.454	—
**CGAN**	75.454	—
**SAGAN**	75.751	—
**GDRCNN-ARM**	1.757	—
**CleanUNet**	46.070	0.00406
**MetricGAN+**	1.895	0.00301
**CMGAN**	1.835	0.04244
**CGAN-SECA (ours)**	75.698	0.00423

As shown in Table 5, the comparison methods differ substantially in parameter count and inference efficiency. Although GDRCNN-ARM, CleanUNet, and MetricGAN+ are more compact, CGAN-SECA achieved higher PESQ and STOI scores in the preceding experiments, demonstrating stronger overall speech-enhancement performance. Its RTF of 0.00423 was also close to those of CleanUNet and MetricGAN+. RTF is defined as the ratio of inference time to the duration of the processed speech, and an RTF below 1 indicates that speech can be processed faster than real time under the tested hardware environment. More importantly, compared with CMGAN, CGAN-SECA reduced the RTF from 0.04244 to 0.00423, corresponding to approximately tenfold faster inference under the same measurement conditions. These results indicate that the relatively large parameter count of CGAN-SECA does not lead to inefficient runtime execution. Nevertheless, with 75.698 million parameters, CGAN-SECA still imposes relatively high storage and memory requirements. Its relatively large parameter count is mainly attributable to the deep symmetric encoder–decoder backbone, in which multiple one-dimensional convolutional and transposed-convolutional layers employ relatively wide feature channels. Future work will investigate reducing the channel width, replacing standard convolutions with depthwise-separable or grouped convolutions, and applying structured pruning, quantization, and knowledge distillation. These strategies are expected to reduce the model complexity and storage requirements while preserving its enhancement performance and inference efficiency.

### Ablation experiment

To further quantify the contribution of each proposed component to the performance of CGAN-SECA, we conducted controlled ablation experiments on the VoiceBank-DEMAND dataset. Starting from the complete CGAN-SECA model, we independently removed the SECA module, VBN, Dropout, and the modified loss function. Group A removed the SECA modules, Group B removed VBN from the discriminator, and Group C removed the Dropout layers. In Group D, the least-squares adversarial objective was replaced with the conventional binary cross-entropy (BCE) objective. All experimental groups used identical training hyperparameters, data preprocessing, and evaluation procedures to minimize the influence of confounding factors. The quantitative results are presented in Table 6, and the corresponding PESQ and STOI trajectories during training are shown in Fig 11.

**Table 6:** Ablation study of the main components of CGAN-SECA on the VoiceBank-DEMAND dataset.

Group	SECA	VBN	Dropout	Modified Loss	PESQ	STOI
Group A	×	√	√	√	2.860	0.943
Group B	√	×	√	√	2.960	0.950
Group C	√	√	×	√	2.991	0.952
Group D	√	√	√	×	2.933	0.948
Full model	√	√	√	√	3.018	0.954

**Fig 11:**
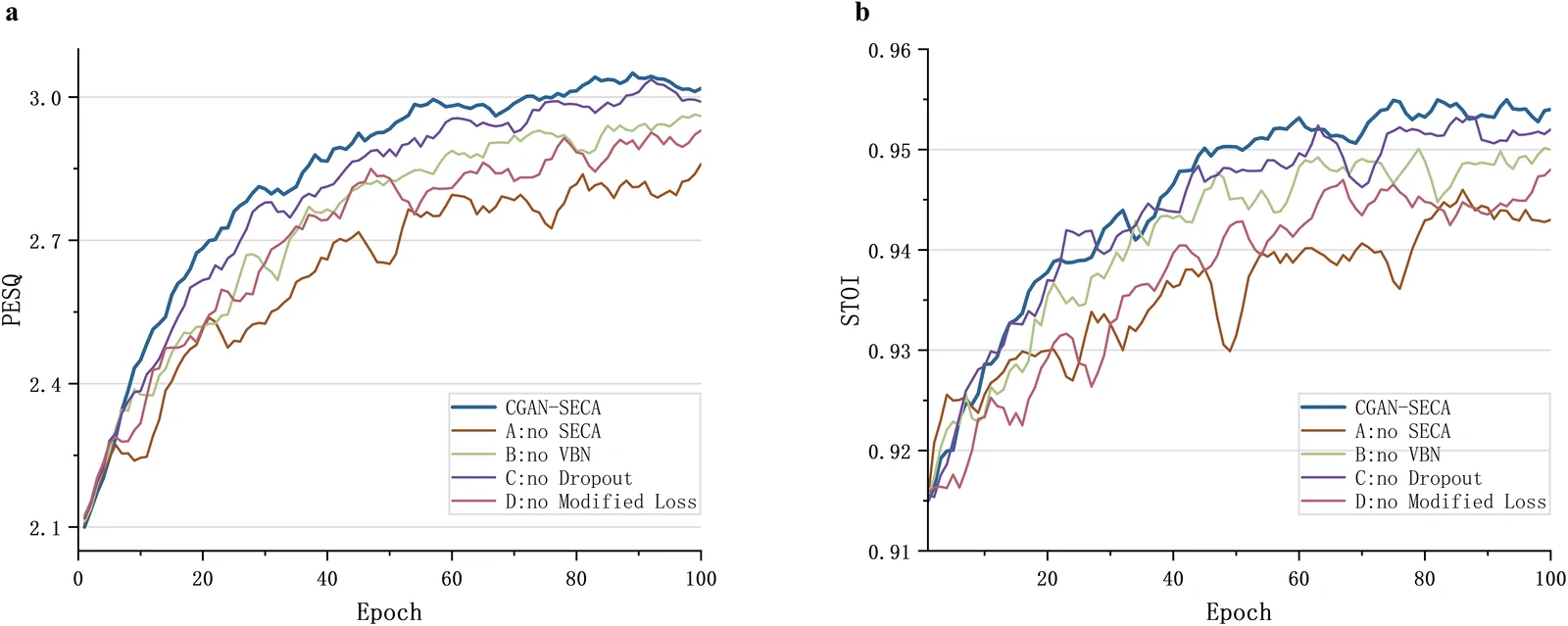
Ablation results of CGAN-SECA during training. (a) PESQ; (b) STOI.

Group A, in which the SECA modules were removed, achieved PESQ and STOI scores of 2.860 and 0.943, corresponding to absolute decreases of 0.158 and 0.011, respectively, compared with the full model. This was the largest performance degradation among all ablation settings. SECA was therefore the most influential component. It uses squeeze-and-excitation operations to adaptively recalibrate the feature channels, assigning greater weights to more informative channels and thereby strengthening speech-relevant responses while suppressing redundant noise related features.

Group B, in which VBN was removed from the discriminator, achieved PESQ and STOI scores of 2.960 and 0.950, corresponding to decreases of 0.058 and 0.004, respectively, compared with the full model. Group C, in which the Dropout layers were removed, achieved PESQ and STOI scores of 2.991 and 0.952, corresponding to decreases of 0.027 and 0.002, respectively. Both effects were smaller than that of removing SECA. Nevertheless, VBN primarily stabilizes feature distributions during discriminator training, whereas Dropout reduces excessive dependence on the training data and thereby mitigates overfitting. Their comparatively modest effects on the final PESQ and STOI scores suggest that, in the proposed network, they mainly support stable adversarial training and model generalization.

Group D replaced the least-squares adversarial loss with the binary cross-entropy (BCE) adversarial loss used in the original CGAN and achieved PESQ and STOI scores of 2.933 and 0.948, corresponding to decreases of 0.085 and 0.006, respectively, compared with the full model. These results indicate that the least-squares adversarial objective is more effective than BCE in improving the perceptual quality and intelligibility of the enhanced speech. This pattern agrees with the loss formulation. The squared-error penalty provides the generator with a continuous optimization signal.

In Fig 11, the horizontal axis represents the training epoch, whereas the vertical axes in panels (a) and (b) represent the validation-set PESQ and STOI scores, respectively. A higher PESQ score indicates better perceptual speech quality, whereas a higher STOI score indicates better speech intelligibility. Removing any individual component reduced both PESQ and STOI to varying degrees. By approximately epoch 12, the complete model already exceeded the model without SECA by 0.223 in PESQ, indicating that SECA improved speech quality from an early stage of training. As training progressed, the performance gaps between the complete model and its ablated variants gradually stabilized. In addition, the models trained without VBN or without the modified loss exhibited more pronounced fluctuations, consistent with the intended roles of VBN and the modified loss in stabilizing adversarial training.

### Statistical significance analysis

To determine whether the performance gains of CGAN-SECA over SAGAN were statistically significant, utterance-level PESQ and STOI scores were calculated for all 824 utterances in the VoiceBank-DEMAND test set. Since both methods were evaluated using the same test utterances, a two-sided paired-sample *t*-test was employed.

First, the variance, standard deviation, and 95% confidence interval (CI) of the PESQ and STOI scores for the two methods were calculated. The results are reported in Tables 7 and 8. It can be observed that CGAN-SECA exhibits slightly lower between-utterance variance and correspondingly narrower confidence intervals for both metrics, indicating less variation in performance across the evaluated utterances.

**Table 7:** Descriptive statistics of PESQ for SAGAN and CGAN-SECA.

PESQ				
Method	Variance	Std	95% CI (lower)	95% CI (upper)
SAGAN	0.5041	0.7100	2.8755	2.9725
CGAN-SECA (ours)	0.4624	0.6800	2.9716	3.0644

**Table 8:** Descriptive statistics of STOI for SAGAN and CGAN-SECA.

STOI				
Method	Variance	Std	95% CI (lower)	95% CI (upper)
SAGAN	0.003600	0.06000	0.93190	0.94010
CGAN-SECA (ours)	0.002704	0.05200	0.95045	0.95755

Let$x_{i}$denote the metric value obtained by SAGAN for thei-thtest utterance, and let${\hat{x}}_{i}$denote the corresponding value obtained by CGAN-SECA. The paired difference is defined as


$$\begin{array}{c} d_{i}={\hat{x}}_{i}-x_{i}, \end{array}$$ (10)

and the test statistic is calculated as


$$\begin{array}{c} t=\frac{\overset{―}{d}}{s_{d}/\sqrt{n}}, \end{array}$$ (11)

where$\overset{―}{d}$is the mean of the paired differences, $S_{d}$is their standard deviation, and n = 824 is the number of paired test utterances. The null hypothesis assumes that the mean paired difference is zero.

As reported in Table 9, the paired comparison yielded t(823)=5.996 for PESQ and t(823)=8.612 for STOI. Both differences were statistically significant. Specifically, CGAN-SECA improved the average PESQ score from 2.924 to 3.018 and the average STOI score from 0.936 to 0.954. Together with the lower variances and narrower confidence intervals, these results indicate that CGAN-SECA provides statistically reliable improvements in perceptual speech quality and intelligibility compared with SAGAN.

**Table 9:** Statistical significance test results.

Metric	*t-value*	*p-value*
PESQ	5.996	3.02 × 10^−9^
STOI	8.612	3.64 × 10^−17^

## Discussion

CGAN-SECA performs waveform-to-waveform speech enhancement using a symmetric encoder–decoder generator with skip connections. This structure transfers multiscale information and reduces the loss of waveform details during downsampling and upsampling. The SECA mechanism adaptively recalibrates channel responses to emphasize speech-related features and suppress noise interference. In the discriminator, VBN and Dropout regularize adversarial training, while the least-squares adversarial objective and L1 reconstruction term jointly improve perceptual quality and waveform fidelity. These components form a coordinated framework for feature extraction, adversarial optimization, and speech reconstruction.

The experimental results demonstrate the strong overall performance of CGAN-SECA. On VoiceBank-DEMAND, it achieved PESQ and STOI scores of 3.018 and 0.954, respectively, outperforming most comparison methods and ranking second among all evaluated methods. On THCHS30-DNS, CGAN-SECA achieved the highest average PESQ and STOI and ranked first for both metrics at 0 and 5 dB. Its RTF of 0.00423 was approximately one tenth of that of CMGAN, demonstrating substantially faster inference. The waveform and spectrogram comparisons further confirmed its ability to suppress broadband noise and preserve speech-dominant temporal and spectral structures. The ablation experiments showed that all four improvements contributed to the final performance, with the removal of SECA causing the largest degradation, followed by the replacement of the least-squares objective with binary cross-entropy. Removing VBN or Dropout also resulted in consistent performance reductions, indicating that channel attention, adversarial optimization, and regularization jointly contribute to the enhancement performance. Furthermore, paired-sample tests on all 824 VoiceBank-DEMAND test utterances confirmed statistically significant improvements over SAGAN in both PESQ and STOI.

Overall, CGAN-SECA achieves a strong balance among enhancement accuracy, robustness under different noise conditions, and inference efficiency. Although its parameter count remains relatively large, this limitation can be addressed through model compression. Future work will further conduct phase-oriented evaluations by comparing CGAN-SECA with representative time–frequency-domain methods under consistent experimental conditions. Circular phase error, group-delay error, and complex spectral distance will be employed to examine the preservation and reconstruction of phase-related speech information.

## Conclusion

This study presented CGAN-SECA, a waveform-domain conditional generative adversarial network that combines a symmetric encoder–decoder generator, SECA-based channel recalibration, VBN and Dropout regularization, and a least-squares adversarial objective with L1 waveform reconstruction. CGAN-SECA achieved PESQ and STOI scores of 3.018 and 0.954 on VoiceBank-DEMAND, respectively, and obtained the highest average scores of 2.274 and 0.838 on THCHS30-DNS while maintaining an RTF of 0.00423. The ablation results verified the contributions of SECA, VBN, Dropout, and the modified loss function, and the paired-sample tests confirmed statistically significant improvements over SAGAN. Overall, these results support the effectiveness and robustness of CGAN-SECA under different speech, noise, and SNR conditions. Future work will focus on reducing the model parameter count and extending the evaluation to phase-related metrics and resource-constrained deployment scenarios.
